# Social Comparison Features in Physical Activity Promotion Apps: Scoping Meta-Review

**DOI:** 10.2196/15642

**Published:** 2020-03-27

**Authors:** Danielle Arigo, Megan M Brown, Kristen Pasko, Jerry Suls

**Affiliations:** 1 Department of Psychology Rowan University Glassboro, NJ United States; 2 Center for Personalized Health Feinstein Institute for Medical Research New York, NY United States

**Keywords:** smartphone app, physical activity, mHealth, social comparison, behavior change technique

## Abstract

**Background:**

Smartphone apps promoting physical activity (PA) are abundant, but few produce substantial and sustained behavior change. Although many PA apps purport to induce users to compare themselves with others (by invoking social comparison processes), improvements in PA and other health behaviors are inconsistent. Existing literature suggests that social comparison may motivate PA for some people under some circumstances. However, 2 aspects of work that apply social comparison theory to PA apps remain unclear: (1) how comparison processes have been operationalized or harnessed in existing PA apps and (2) whether incorporating sources of variability in response to comparison have been used to tailor comparison features of apps, which could improve their effectiveness for promoting PA.

**Objective:**

The aim of this meta-review was to summarize existing systematic, quantitative, and narrative reviews of behavior change techniques in PA apps, with an emphasis on social comparison features, to examine how social comparison is operationalized and implemented.

**Methods:**

We searched PubMed, Web of Science, and PsycINFO for reviews of PA smartphone apps. Of the 3743 initial articles returned, 26 reviews met the inclusion criteria. Two independent raters extracted the data from these reviews, including the definition of social comparison used to categorize app features, the percentage of apps categorized as inducing comparison, specific features intended to induce comparison, and any mention of tailoring comparison features. For reference, these data were also extracted for related processes (such as behavioral modeling, norm referencing, and social networking).

**Results:**

Of the included review articles, 31% (8/26) categorized app features as prompting social comparison. The majority of these employed Abraham and Michie’s earliest definition of comparison, which differs from versions in later iterations of the same taxonomy. Very few reviews specified what dimension users were expected to compare (eg, steps, physical fitness) or which features of the apps were used to induce comparison (eg, leaderboards, message boards). No review referenced tailoring of comparison features. In contrast, 54% (14/26) reviews categorized features for prompting behavioral modeling and 31% (8/26) referenced tailoring app features for users’ personal goals or preferences.

**Conclusions:**

The heterogeneity across reviews of PA apps and the absence of relevant information (eg, about dimensions or features relevant for comparison) create confusion about how to best harness social comparison to increase PA and its effectiveness in future research. No evidence was found that important findings from the broader social comparison literature (eg, that people have differing preferences for and responses to social comparison information) have been incorporated in the design of existing PA apps. Greater integration of the mobile health (mHealth) and social comparison literatures may improve the effectiveness of PA apps, thereby increasing the public health impact of these mHealth tools.

**International Registered Report Identifier (IRRID):**

RR2-https://osf.io/nh4td/

## Introduction

Despite decades of intervention efforts by several health care disciplines, physical inactivity remains a leading cause of morbidity and mortality in the United States [1]. Many emerging digital health interventions focus on promoting physical activity (PA) [2], delivered via mobile health (mHealth) applications or smartphone apps. For example, more than 5000 apps available from the iTunes and Google Play app stores are designed to promote PA (alone or in the context of weight loss) [3]. Although many of these apps are user-friendly and elicit high user engagement [4], most are designed without input from behavioral scientists or other health professionals and reach the market without rigorous scientific evaluation [5,6]. Conversely, evidence-based PA apps have been developed by researchers, but these apps rarely reach the commercialization stage (due to a lack of resources) and research participants show modest engagement with them [7]. These limitations may contribute to the low efficacy of existing PA apps; those that have been tested in randomized controlled trials produce only short-term increases in activity [8].

Thus, few existing PA apps are simultaneously grounded in behavior change science, engaging for potential users, and effective over the long term. Efforts are needed to improve PA app design to optimize both user engagement and intervention effectiveness. Currently, both commercial and researcher-developed PA apps vary in the extent to which they employ specific behavior change techniques (BCTs) [9]. In fact, considerable research effort has been devoted to determining the number and type of BCTs in existing apps. *Social comparison* (ie, evaluating one’s standing relative to others) [10] is a BCT used in several commercial and researcher-developed apps [6]. Comparison has also been identified as one of the most effective techniques for promoting PA in face-to-face behavioral interventions [11,12]. In PA apps, social comparison is activated when a user’s information is listed alongside that of other users, for example, via activity engagement rankings (leaderboards). Comparison may also be activated by any feature that exposes app users to information about other users (eg, message boards or other social networking features). However, PA app developers have not always recognized that social comparison is a complex process; it can be activated by various factors and has several possible outcomes. A comprehensive assessment of how social comparison is being currently used in PA apps and whether current methods capitalize fully on the theoretical and empirical social comparison literature has not been available. Such a review could begin to suggest how to optimize an app’s social comparison features and, potentially, improve its efficacy.

To illustrate the complexities of social comparison processes, consider that PA is a multifaceted concept; there are various *dimensions* of PA (eg, steps per day, minutes of intense aerobic activity per week, appearance of muscularity, overall physical fitness), and app users may focus on any or all of these as the subject of social comparison. In addition, BCTs such as behavioral modeling (ie, providing examples of behavior engagement to encourage others to engage) and norm referencing (ie, providing information about group norms or averages) often are differentiated from social comparison as mechanisms of behavior change [9]. However, these mechanisms can explicitly or implicitly prompt a comparison of an aspect of the self to another person (or persons). Furthermore, modeling and norm referencing are assumed to prompt social comparisons in some classification systems [13]. An additional complication is that although research has found that social comparisons (via leaderboards or through these other processes) may promote PA [14,15], some experiments find that social comparisons can have negative consequences, such as worsened mood and decreased motivation for or engagement in healthy behavior [16-19]. Exposing users to others who have engaged in more PA than they have might be either inspiring (by learning what they might achieve [20]) or discouraging (by seeing themselves as inferior or incapable of achieving activity goals [16,21,22]). Conversely, exposing users to others who have engaged in less PA than they have may be satisfying (because they are outperforming their peers) or stressful (because they might also become more sedentary) [23,24].

Moreover, existing literature on social comparison processes shows that people’s responses to comparison, as well as their preferences for the comparison information they receive, differ at 2 levels. At the between-person (or dispositional) level, different users may show different responses or preferences that are consistent over time [25]. At the within-person level, the same user may show variability in their responses and preferences over time [26,27]. Devising apps to modify social comparison features to match the general preferences of individual users or contextual preferences over time might be more effective for promoting PA, versus exposing everyone to the same comparison information. Such personalization or tailoring may prevent users from disengaging from social comparison or from PA apps altogether, especially if they repeatedly receive (potentially) discouraging comparison information [16,28].

To what extent distinct dimensions and possible outcomes of social comparison are considered in existing PA apps remains an open question. A search of available literature reveals more than 100 published reviews about PA apps, surveying thousands of individual app-based programs. A number of these reviews intentionally categorize app features, including social comparison (using the BCT taxonomy [9] and other frameworks). These summaries are intended to inform future app design and evaluation [29,30]. However, to our knowledge, no review or synthesis of reviews has focused on social comparison or considered whether findings from the mainstream comparison literature have been incorporated.

This scoping review had the following objectives: (1) to determine how social comparison is currently defined and categorized in existing systematic, meta-analytic, and narrative reviews of commercially available and researcher-developed PA apps, (2) to examine the methods for activating and facilitating social comparison in the PA apps identified in these reviews, and (3) to determine to what extent different elements of social comparison are included as design features in the PA apps. This review represents an initial step for the formulation of best practice recommendations for including social comparison features in PA apps.

## Methods

### Guidelines

This review followed the initial guidelines delineated by Arksey and O’Malley [31] and the recent Preferred Reporting Items for Systematic Reviews and Meta-Analyses (PRISMA) Extension for Scoping Reviews (PRISMA-ScR) [32]. A description of the protocol for this review is registered with the Open Science Framework.

### Research Questions

This scoping review was guided by the following a priori research questions:

1. How often does social comparison appear as a key behavior change mechanism in published reviews of PA smartphone apps?
2. How is social comparison defined in published reviews of PA smartphone apps?
3. How are app features categorized as social comparison (vs other behavior change processes) in published reviews of PA apps?
4. What methods by which social comparison is activated or facilitated in PA apps are included in published reviews?
5. To what extent (and how) have PA apps included in published reviews addressed between- and within-person variability in responses to social comparison (eg, via tailoring)?
6. To what extent (and how) is social comparison differentiated from related processes, such as modeling and norm referencing, in published reviews of PA apps?

How effective social comparison features of apps are in changing PA behavior is also an important question. It is not included in the preceding list because we did not find any randomized controlled trials, narrative reviews, meta-analyses, or dismantling studies focused on social comparison app features or directly comparing the effects of different app features. We elaborate on this point in the *Discussion* section.

### Identification and Selection of Relevant Reviews

Inclusion and exclusion criteria were chosen by the first and last authors (DA and JS). Review articles were eligible for inclusion if they met the following criteria: (1) available in English; (2) published on or before May 31, 2019; (3) conducted a systematic or narrative review, or meta-analysis; (4) reviewed the features of commercially available smartphone apps or included formal intervention programs delivered via smartphone apps; and (5) used increasing PA or reducing sedentary time as a key behavioral outcome. An initial examination of the literature revealed that many reviews in the domain of mHealth combine PA with related weight control outcomes. Consequently, reviews that met the first 4 criteria and used weight loss or PA plus other behaviors (eg, diet and weight loss) as outcomes were included.

Reviews were excluded if they considered interventions that combined an app modality with other modalities (eg, websites, text messages, etc) because they might obscure conclusions specific to apps. Apps geared toward particular medical populations also were excluded because these interventions tend to promote multiple behavior changes and set illness-specific PA targets, rather than focusing on broad-based PA increases. Finally, apps involving gamified interventions were excluded because they typically engage a variety of social processes, in addition to social comparison, to create competition with other users or teams of users. Dissecting comparison features from features intended to induce other processes in this context can be very challenging.

**Figure 1:**
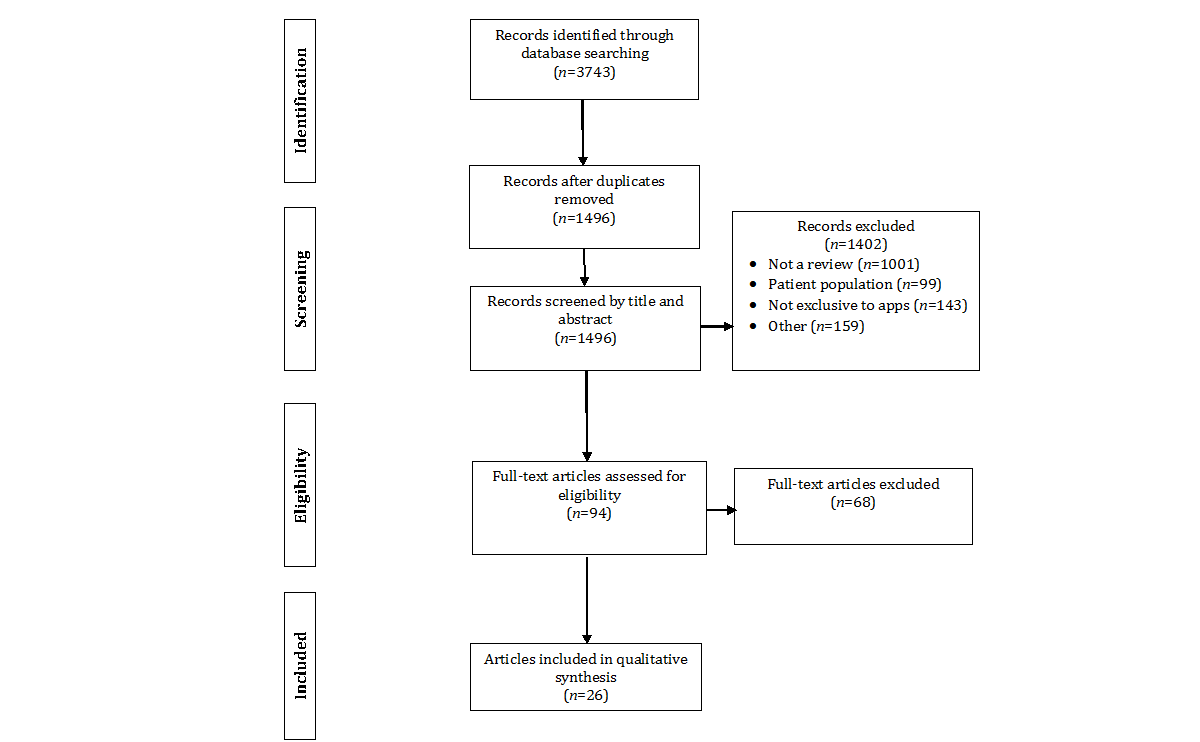
Preferred Reporting Items for Systematic Reviews and Meta-Analyses (PRISMA) Extension for Scoping Reviews flowchart.

We searched PubMed, PsycINFO, and Web of Science for publications related to the use of smartphone apps for increasing PA. Search terms were combinations of “physical activity” or “exercise” and “smartphone app(lication),” “mobile app(lication),” or “mHealth.” Resulting titles and abstracts were reviewed to determine relevance to our 6 research questions. Initial database and hand searches returned 3743 individual articles of which 2247 were duplicates, leaving 1496 unique articles. A PRISMA-ScR flowchart, shown in Figure 1, details the evaluation of each article for inclusion in this review. The majority of articles that were identified described empirical studies. Initial reviews were conducted by the first 3 authors (DA, MB, and KP) who were responsible for determining inclusion for an equal subset of identified articles. Final review and inclusion decisions were made by the first author (DA). The final set of 26 review articles were coded for the characteristics described in the following section.

### Data Extraction and Article Coding

The first and last authors (DA and JS) determined the types of data to be extracted from each article. The second and third authors (MB and KP, respectively) each independently read and extracted the following data from the 26 reviews: authors; year of publication; review of commercially available versus researcher-developed apps (or combination); number of apps reviewed; specific behavior change outcome targeted by the app (eg, overall PA, sedentary behavior, weight loss); percentage of apps that included social comparison features; the definition of social comparison; the specific features for inducing social comparison (eg, leaderboards); the social comparison dimension (eg, steps, physical fitness); and the presence (vs absence) and type or types of social comparison tailoring. Additional data extracted included the percentage of apps categorized as modeling/demonstrating a behavior, providing normative information about others' behavior, and social networking (eg, message boards). These features are associated with the opportunity to make comparisons, even if comparison is not considered the primary BCT induced.

For reviews that explicitly categorized features based on social comparison or other types of social influence (eg, modeling), the percentages attributed to social comparison processes were taken directly from the original published review. For reviews that did not use these terms, the percentages were calculated manually by reviewing the details available in the original published review, where possible (eg, references to social networking features or exposure to information about other users). As for all other data extraction, the second and third authors (MB and KP, respectively) independently determined the percentages of apps that categorized features as inducing social comparison or other social processes. The first author (DA) then calculated the interrater agreement (91%) and independently rated a subset of included reviews to verify the accuracy; the remaining discrepancies were resolved by consensus.

## Results

### Types of Reviews

Among the 26 articles reviewed, the number of apps identified as promoting PA or weight control ranged from 12 [33] to more than 28,000 [34]. Of these 26 articles, 10 (38%) focused exclusively on apps intended to increase PA and 10 (38%) focused on weight loss, weight management, or obesity intervention (the largest subsets; see Table 1). The remaining reviews (6/26, 23%) considered a combination of diet, PA, and/or weight control/obesity prevention. The majority of articles reviewed only commercially available apps (19/26, 73%), primarily those available through the iTunes App Store (Apple operating system). Only 27% (7/26) of reviews appeared to include apps developed or empirically tested by researchers. The popularity of these reviews appeared to increase through 2014 (peaking in 2014-2016) and then decrease through 2019.

**Table 1:** Descriptive information for each included review of physical activity and related apps.

Author and year	Type(s) of apps	Apps reviewed, n	Operating system(s)	Behavioral target or targets
Azar et al (2013) [35]	Commercial	23	Apple	Weight management
Bardus et al (2016) [4]	Commercial	23	Apple and Android	Weight management
Bondaronek et al (2018) [36]	Commercial	65	Apple and Android	Increase physical activity
Brannon and Cushing (2015) [37]	Both^a^	200 (number of PA apps only)	Apple	Improve diet or physical activity
Breton et al (2011) [38]	Commercial	204	Apple	Weight management
Conroy et al (2014) [29]	Commercial	200	Apple and Android	Increase physical activity
Direito et al (2014) [39]	Commercial	40	Apple	Weight management
Dute et al (2016) [33]	Researcher developed	12	Apple	Healthy nutrition, or physical activity, or overweight prevention
Jee (2017) [40]	Both	200	Apple and Android	Increase physical activity
Jeon et al (2014) [41]	Commercial	104	Apple	Weight management
Knight et al (2015) [30]	Commercial	379	Apple and Android	Increase physical activity
Middelweerd et al (2014) [3]	Commercial	64	Apple and Android	Increase physical activity
Modave et al (2015) [42]	Commercial	30	Apple	Increase physical activity
Nikolaou and Lean (2017) [34]	Commercial	28,905	Apple, Android, Amazon, Windows, Blackberry	Weight management
Payne et al (2015) [43]	Commercial	52	Apple	Increase physical activity
Quelly et al (2016) [44]	Researcher developed	9	Not applicable	Improve weight-related behaviors, psychosocial factors, and/or anthropometric outcomes
Rivera et al (2016) [5]	Commercial	393	Apple, Android, Blackberry, Windows	Weight management
Schoeppe et al (2016) [45]	Both	27	Not mentioned	Improve diet, physical activity, and/or sedentary behavior
Schoeppe et al (2017) [46]	Commercial	25	Apple and Android	Improve diet, physical activity, and/or sedentary behavior
Schoffman et al (2013) [47]	Commercial	57	Apple	Pediatric weight management
Stuckey et al (2017) [48]	Both	18	Not mentioned	Increase physical activity
Vlahu-Gjorgievska et al (2018) [49]	Researcher tested^b^	6	Not mentioned	Weight management
Wang et al (2015) [50]	Commercial	10	Android	Increase physical activity
Wearing et al (2014) [51]	Commercial	62	Apple	Pediatric weight management
West et al (2012) [52]	Commercial	3336	Apple	Increase physical activity
Yang et al (2015) [53]	Commercial	100	Apple and Android	Increase physical activity

^a^Both: the article reviewed both commercially available and researcher-developed apps.

^b^Researcher tested: commercially available apps evaluated in formal research studies.

### Social Comparison in Physical Activity Apps

#### Reference to Social Comparison as a Behavior Change Mechanism

Of the included review articles, 31% (8/26) categorized app features as inducing social comparison (see Table 2). The percentages of apps with social comparison features ranged from 8% (2/27) [45] to 66% (43/65) [36], with an average of 30% across reviews that used social comparison as a category (see Table 3).

**Table 2:** Summary of social comparison data extracted from each review of physical activity apps.

Author and year	SC^a^ definition	Apps with SC as a category, n (%)	SC features	Apps specifying the SC dimension, n (%)	Apps with SC tailoring, n (%)
Azar et al (2013) [35]	Not mentioned	Not mentioned	Not mentioned	Not mentioned	Not mentioned
Bardus et al (2016) [4]	Not mentioned	Not mentioned	Not mentioned	Not mentioned	Not mentioned
Bondaronek et al (2018) [36]	Michie et al (2013) [13]	43 (66)^b^	Not mentioned	43 (66)^b^ (comparison of behavior)	Not mentioned
Brannon and Cushing (2015) [37]	Abraham and Michie (2008) [9]	9 (14)^c^	“Most commonly be seen in the case of group practice but could also be employed using detailed case studies in text or video or by pairing people as supports.”	Not mentioned	Not mentioned
Breton et al (2011) [38]	Not mentioned	Not mentioned	Not mentioned	Not mentioned	Not mentioned
Conroy et al (2014) [29]	Michie et al (2011) [54]	25 (15.0)^d^	Not mentioned	Not mentioned	Not mentioned
Direito et al (2014) [39]	Abraham and Michie (2008) [9]	22 (55)^e^	Not mentioned	Not mentioned	Not mentioned
Dute et al (2016) [33]	Abraham and Michie (2008) [9]	1 (8)^f^	Activity sharing and connection to a partner whose activities are visible	4 (33)^f^ (sharing activities)	Not mentioned
Jee (2017) [40]	Not mentioned	Not mentioned	Competitions	Not specified	Not mentioned
Jeon et al (2014) [41]	Not mentioned	Not mentioned	Not mentioned	Not mentioned	Not mentioned
Knight et al (2015) [30]	Not mentioned	Not mentioned	Not mentioned	Not mentioned	Not mentioned
Middelweerd et al (2014) [3]	Abraham and Michie (2008) [9]	10 (16)^g^	Not mentioned	Not mentioned	Not mentioned
Modave et al (2015) [42]	Not mentioned	Not mentioned	Not mentioned	Not mentioned	Not mentioned
Nikolaou and Lean (2017) [34]	Not mentioned	Not mentioned	Not mentioned	Not mentioned	Not mentioned
Payne et al (2015) [43]	Not mentioned	Not mentioned	Not mentioned	Not mentioned	Not mentioned
Quelly et al (2016) [44]	Not mentioned	Not mentioned	Not mentioned	Not mentioned	Not mentioned
Rivera et al (2016) [5]	Not mentioned	Not mentioned	Not mentioned	Not mentioned	Not mentioned
Schoeppe et al (2016) [45]	Not mentioned	Not mentioned	Social comparison with friends via leaderboards	Not specified (comparison of *physical activity behavior*)	Not mentioned
Schoeppe et al (2017) [46]	Abraham and Michie (2008) [9]	10 (40)^h^	Competitions, leaderboards	Not specified	Not mentioned
Schoffman et al (2013) [47]	Not mentioned	Not mentioned	Not mentioned	Not mentioned	Not mentioned
Stuckey et al (2017) [48]	Not mentioned	Not mentioned	Not mentioned	Not mentioned	Not mentioned
Vlahu-Gjorgievska et al (2018) [49]	Not mentioned	Not mentioned	Not mentioned	Not mentioned	Not mentioned
Wang et al (2015) [50]	Not mentioned	Not mentioned	Not mentioned	Not mentioned	Not mentioned
Wearing et al (2014) [51]	Not mentioned	Not mentioned	Not mentioned	Not mentioned	Not mentioned
West et al (2012) [52]	Not mentioned	Not mentioned	Not mentioned	Not mentioned	Not mentioned
Yang et al (2015) [53]	Michie et al (2013) [13]	25 (25.0)^i^	Not specified	Not specified	Not mentioned

^a^SC: social comparison.

^b^N=65.

^c^N=66.

^d^N=167.

^e^N=40.

^f^N=12.

^g^N=64.

^h^N=25.

^i^N=100.

**Table 3:** Percentages of articles reviewed (26 articles) that included each behavior change technique (BCT) category, and average percentages of apps identified by these articles as including features that belong to each BCT category.

Behavior change technique (BCT)	Percentage of articles reviewed (N=26)	Average percent of apps with designated features
Social comparison	31	30
Modeling	54	35
Normative feedback	12	13
Social networking	38	32
Tailoring (general)	31	40

#### Definitions of Social Comparison

The majority of articles that referenced social comparison (5/8, 63%) employed Abraham and Michie’s [9] BCT definition of social comparison—“facilitate[ing] observation of nonexpert others’ performance for example, in a group class or using video or case study.” Other definitions included those proposed by Michie et al’s [54] revised Coventry, Aberdeen & London – Refined CALO-RE BCT or Michie et al’s [13] hierarchy of BCTs; see Table 4 for the full text and frequencies of these definitions. Of note, Abraham and Michie’s [9] definition specifies that comparison targets are *nonexperts*, and Michie et al’s [54] definition explicitly states that merely exposing users to others using group settings does not constitute social comparison, as several other processes could be engaged (eg, modeling, social support).

**Table 4:** Definitions of social comparison used in existing reviews of physical activity apps.

Author and year	Definition	Reviews using this definition, n (%)^a^
Abraham and Michie (2008) [9]	“Facilitate observation of nonexpert others’ performance for example, in a group class or using video or case study.”	6 (67)
Michie et al (2011) [54]	“Facilitate social comparison Involves explicitly drawing attention to others’ performance to elicit comparisons. NB: The fact the intervention takes place in a group setting, or have been placed in groups on the basis of shared characteristics, does not necessarily mean social comparison is actually taking place. Social support may also be encouraged in such settings. Group classes may also involve instruction, demonstration, and practice.”	1 (11)
Michie et al (2013) [13]	“Draw attention to others’ performance to allow comparison with the person’s own performance. *Note:* being in a group setting does not necessarily mean that social comparison is actually taking place.”	2 (22)

^a^Percentages above use a denominator of N=8, the number of reviews that categorized app features as social comparison.

#### Social Comparison App Features

Across definitions, only some of the articles that categorized social comparison (5/8, 63%) specified or implied which features they considered to induce comparison. These reviews referenced *leaderboards* [46], *competitions* [40], *sharing information with other users* [33], and *connections between users* [30]. One article described social comparison as features such as “group practice… [and] detailed case studies in text or video or by pairing people as supports” [37]. Another review indicated that *friendly competitions* were available in some apps but *did not* include them as features that prompt social comparison [45].

#### Dimension of Comparison

Of the 8 articles that categorized features inducing social comparison, 3 (38%) referenced the specific dimension. One review indicated that users could share/compare *their activities* (33% of apps reviewed) [33]; the other distinguished between apps that allowed for *comparison of behavior* (66% of apps reviewed) and *comparison of outcomes* (13% of apps reviewed) [36]. *Comparison of behavior* was most often described as a demonstration of particular exercises (ie, modeling), whereas *comparison of outcomes* referred to potential consequences of a behavior, rather than to social comparison [13]. The third review described apps that allowed sharing/comparing *PA information* [46], although without specifying the percentages of apps with such features.

#### Acknowledgment of Between- and Within-Person Variability or Tailoring of Comparison Features

None of the articles reviewed referred to individual (between-person) differences in social comparison responses or preferences, a change in these responses or preferences (within-person) over time, or tailoring social comparison features to address either level of variability. In contrast, 8 of the 26 included articles (31%) described tailoring or personalization with respect to feedback on user progress toward behavioral goals (92% of apps reviewed; see Table 5) [36]. For example, users who did not meet the PA guidelines for a given period were given a visual comparison of their PA to the recommended level of PA (vs reinforcement for those who met the guidelines), with PA information matched to users’ demographic characteristics (eg, PA and aging for those over 45, PA and weight loss for those with BMIs greater than 25) [55]. Reviews also referenced tailoring with respect to matching *motivational cueing* (28% of apps reviewed) [48], *exercise prescriptions* (11% of apps reviewed) [48], and *encouraging messages* (33% of apps reviewed) [49] to users’ progress and/or preferences.

**Table 5:** Summary of tailoring, modeling, norm referencing, and social networking data extracted from each review of physical activity apps.

Author and year	Apps with any tailoring, n/N (%)	Apps with modeling as a category, n/N (%)	Apps with normative feedback, n/N (%)	Apps with social networking capabilities, n/N (%)
Azar et al (2013) [35]	Not mentioned	Not specified	Not mentioned	Not mentioned
Bardus et al (2016) [4]	Not mentioned	Not mentioned	Not mentioned	3.45/23 (15)
Bondaronek et al (2018) [36]	60/65 (92)	31/65 (47)	Not mentioned	Not mentioned
Brannon and Cushing (2015) [37]	Not mentioned	124/200 (62.0)	Not mentioned	Not mentioned
Breton et al (2011) [38]	Not mentioned	Not mentioned	Not mentioned	7/204 (3.4)
Conroy et al (2014) [29]	Not mentioned	106/200 (53.0)	2/200 (1.0)	Not mentioned
Direito et al (2014) [39]	Not mentioned	21/40 (53)	Not mentioned	Not mentioned
Dute et al (2016) [33]	4/12 (33)	4/12 (33)	Not mentioned	3/12 (25)
Jee (2017) [40]	Not mentioned	Not mentioned	Not mentioned	Not mentioned
Jeon et al (2014) [41]	Not mentioned	Not mentioned	Not mentioned	Not mentioned
Knight et al (2015) [30]	Not mentioned	Not mentioned	Not mentioned	209/379 (55.1)
Middelweerd et al (2014) [3]	64/64 (100)	7/64 (11)	Not mentioned	Not mentioned
Modave et al (2015) [42]	Not mentioned	Not mentioned	Not mentioned	1/30 (3)
Nikolaou and Lean (2017) [34]	Not mentioned	Not mentioned	Not mentioned	Not mentioned
Payne et al (2015) [43]	Not mentioned	16/52 (31)	3/52 (6)	Not mentioned
Quelly et al (2016) [44]	3/9 (33)	1/9 (11)	3/9 (33)	Not mentioned
Rivera et al (2016) [5]	7/393 (1.8)	Not mentioned	Not mentioned	Not mentioned
Schoeppe et al (2016) [45]	7/27 (26)	2/27 (7)	Not mentioned	4/27 (15)
Schoeppe et al (2017) [46]	Not mentioned	6.75/25 (27)	Not mentioned	Not mentioned
Schoffman et al (2013) [47]	5/57 (9)	Not mentioned	Not mentioned	9/57 (16)
Stuckey et al (2017) [48]	5/18 (28)	Not mentioned	Not mentioned	Not mentioned
Vlahu-Gjorgievska et al (2018) [49]	Not mentioned	Not mentioned	Not mentioned	Not mentioned
Wang et al (2015) [50]	Not mentioned	3.7/10 (37)	Not mentioned	7.8/10 (78)
Wearing et al (2014) [51]	Not mentioned	18/62 (29)	Not mentioned	Not mentioned
West et al (2012) [52]	Not mentioned	1235/3336 (37.0)	Not mentioned	1535/3336 (46.0)
Yang et al (2015) [53]	Not mentioned	47/100 (47.0)	Not mentioned	32/100 (32.0)

### Other App Processes Relevant to Social Comparison

#### Modeling/Demonstrating Behavior

Of the 26 articles, 14 (54%) classified app features as modeling or demonstrating particular behaviors (eg, proper exercise form; see Table 5). The percentage of app features categorized as modeling in each review ranged in size from 7% [45] to 53% [29,38], with an average of 35%. One review indicated that modeling was a popular BCT but did not specify the percentage of apps with this feature [35]. Behavioral models were either fitness professionals (coaches) or app users who appeared via a photo or video. Although these features were not counted as inducing comparison, modeling represents an attempt to increase similarity (or decrease the perceived difference) between the app user's behavior and a comparison target's behavior. Consequently, modeling features may facilitate social comparison.

#### Normative Feedback

Providing normative information about others’ behavior is intended to give an individual user a sense of how they compare to the average for a relevant group. Although social comparison often refers to comparisons against individual targets, comparison to a group average is a related process [56]. Of the 26 articles, 3 (12%) evaluated whether apps provided normative information to users. These articles reported that normative information appeared in 1% [29] to 33% [44] of the apps reviewed, with an average of 13%.

#### Social Networking

Of the 26 articles, 10 (38%) referenced social networking features via app-specific communities or connections to existing social media platforms. Percentages of apps designated as offering these features ranged from 3% [38,42] to 78% [50], with an average of 32%. Although social networking platforms can facilitate several social influence processes (eg, social reinforcement or support), social comparisons between users of these platforms are common (based on shared text, objective data, or images) and are associated with a range of affective and behavioral responses [57,58].

## Discussion

### Reviewing Evidence of Social Comparison in Physical Activity Apps

Social comparison is known to influence motivation and health behavior and is frequently manipulated in health behavior change interventions [9]. Comparison processes may be particularly useful for promoting PA with technology such as smartphone apps; objective measures of PA can be visualized and shared between app users, and users can see evidence of change in their relative standing by increasing their PA behavior over short time frames. Despite the interest in social comparison as a motivator of PA change and the exponential increase in publications about digital health interventions [59], no review to date has attempted to summarize existing literature on the social comparison features of PA apps. We undertook the present scoping review to address this gap and provide recommendations for future research in this area.

### Defining and Classifying Social Comparison

A modest proportion of the 26 available and eligible reviews of PA promotion apps categorized app features as eliciting social comparison (31%). Comparison fell behind modeling as a popular intervention process (54%) but was as common as social networking (38%; which also may facilitate comparison) and was more common than related processes such as norm referencing (12%). All the articles that included social comparison as a category used versions of the BCT taxonomy [9,13,54]. However, the versions differ in their definitions of social comparison. The original BCT taxonomy specifies that the potential target of comparison must be a *nonexpert* [9]; exposure to an expert is classified as modeling. Although modeling appeared more frequently in apps than did social comparison, the percentages of apps with features in each category differed modestly (ie, 35% vs 30%; see Table 3). Later iterations of the BCT taxonomy removed the requirement that only social comparisons with nonexperts would qualify [13,54]. Visual inspection of the percentage of apps classified as having social comparison features suggests that using the broader definition, ie, including experts, slightly increases the average proportions of apps that receive a social comparison designation (ie, 27% to 35%). The broader definition also is consistent with definitions of social comparison used in the mainstream comparison literature, where targets often include media figures or fashion models, in addition to peers [60].

Abraham and Michie’s [9] initial taxonomy also defined comparison as simply *observation* of another’s performance, which may occur in a variety of contexts (eg, group classes). Using this definition, PA app features such as social networking or message boards (where users can report on their performance) may count as social comparison [30,33]. In contrast, later versions explicitly state that attention must be drawn to the other’s performance and that contexts such as group classes do not necessarily induce comparison (vs other social processes) [13,54]. This definition implies that social networking and message boards would not count as social comparison, whereas leaderboards or competitions would [40,46].

The majority of reviews did not include any mention of specific dimensions of social comparison, and those that did made only vague references to dimensions (eg, *comparison of behavior* without specifying which behavior, eg, steps, etc). A recent meta-analysis suggests that comparison dimension provides information about the target’s relevance to the self; if relevance to the self is not clear, the individual might reflect on their target’s performance but not engage in comparative self-evaluation [61]. Owing to the many dimensions potentially relevant to PA promotion (eg, steps, calories burned, minutes of activity, and overall fitness) and the likelihood that these dimensions are not relevant for all app users [62], this review highlights the need for increased specificity in future work that describes social comparison features of apps.

As very few articles included descriptions of the specific features eliciting comparison, the exact degree of heterogeneity is unclear. What can be concluded is that existing reviews of PA apps show considerable variability in their approaches to defining and classifying social comparison. Specifically, comparison, modeling, and information sharing are not consistently differentiated. The heterogeneity associated with which features activate social comparison represents a challenge for future research to evaluate the unique effect of comparison as a mechanism of app-based behavior change, or its efficacy relative to other mechanisms [15]. Inconsistency in the definition of comparison also creates challenges for optimizing app-based interventions to address comparison preferences and needs between users, which may be either stable or dynamic. In this vein, PA app development has not yet integrated theoretical and empirical advances that the mainstream social comparison literature has made.

### Social Comparison Theory and Evidence Relevant to Physical Activity App Design

Interest in and responsiveness to social comparison information vary across individuals. This construct, called social comparison orientation (SCO) [63], has been positively associated with engagement in PA [64]. PA app users with strong SCOs may engage in comparison in response to a wide variety of social features in PA apps, including social networking and message boards, and they may find this information motivating. Here, comparison information is available, but the comparison process itself is not intentionally activated. In contrast, users with weaker SCOs may engage in comparison only when the comparison process is deliberately induced, such as by competitive challenges or leaderboards that display PA data ranked from most to least [65]. Social comparison features also may be ineffective for users with weaker SCOs. These hypotheses imply that PA app effectiveness might be improved by guiding users toward the types of social features that match their level of SCO or away from social comparison features at particularly low levels of SCO.

Additional variability may exist with respect to users’ social comparison preferences and their affective and behavioral responses to comparisons. As noted, users may find comparisons to targets who are *doing better* with respect to PA (ie, upward comparisons) either inspiring or disheartening and may find comparisons to targets who are *doing worse* (ie, downward comparisons) either comforting or anxiety-provoking [18,23]. Which combinations lead to the greatest increases in PA (or lead to increases vs decreases) and for whom are significant empirical questions [25,66,67]. Basic research indicates that the opportunity to select a comparison target does not always lead to optimal affective or health-relevant outcomes, nor does it always fulfill comparers’ goals (eg, *to feel better*) [18,68,69]. Thus, providing information about only the targets that a PA app user wants may not lead to benefits. Providing only the targets that they do *not* want may create an aversive experience, however, and may lead users to discontinue engagement with the app [28].

The optimal combination of comparison target and affective response for increasing PA may differ between people. The best combination may also vary within the same person over time, as a function context (eg, precomparison mood), shift over the course of behavior change (eg, as users experience progress and setbacks) [56,70], and differ from users’ stated preferences, depending on whether users are just starting with the app or have been engaged for some time. The degree of within-person variability in social comparison preference and response (either affective or behavioral) remains unclear. The quantification of within-person variability and its responsiveness to social comparison interventions (eg, using N-of-1 designs) represent important next steps for PA app development and a broader understanding of social comparison processes [71].

### Future Directions for Social Comparison Features of Physical Activity Apps: Social Comparison Tailoring

Despite gaps in the social comparison literature, evidence suggests that the effects of social comparison and preferences for a comparison type differ between people and within people over time. This review, however, detected no reference to between- or within-person variability in comparison response/preference or to tailoring social comparison features of PA apps. In contrast, this review indicates that tailoring in PA apps is common with respect to goals and feedback, which suggests that technology for such tailoring is currently in use. Tailoring the PA app experience to match user characteristics such as SCO or user-relevant PA comparison dimensions might improve the app’s acceptability and engagement and, in turn, enhance PA outcomes [28]. Indeed, tailoring has been shown to outperform generic messaging in PA interventions across a range of modalities, including apps [48,72]. Tailoring also might discourage negative consequences of comparison (eg, giving up in response to a failure to match another user's achievements) by matching a user’s comparison preferences with the types of comparisons that optimize engagement in PA. Such tailoring will require nuanced assessment of the effect of factors such as SCO, dimensions of relevance, comparison preferences, affective response to comparison, and PA engagement. The adaptive capabilities of many existing apps and those under development may lend themselves to such tailoring [73].

### Strengths, Limitations, and Additional Future Directions

Strengths of this scoping review include its use of preregistered methods, adherence to PRISMA-ScR guidelines, and a comprehensive search for relevant reviews to provide insights into how social comparison is currently applied in existing PA apps. A subset of pertinent articles may have been overlooked, but the extensive and systematic search increases confidence in the overall conclusions. Additional app comparison features (eg, specific dimensions and tailoring) may have not been described in the reviews or missed by our coders. As a check, we examined several primary sources of empirical data and failed to find these additional details. One exception, an empirical study by Mollee and Klein [28], demonstrated PA benefits of matching (tailoring) versus not matching comparison targets to user preferences. There is need for additional work of this kind to inform best practices for tailoring social comparison features of PA apps.

Although social comparison has been shown as effective for increasing PA in other types of interventions (eg, team-based competitions) [26], there are very few studies of the effectiveness of social comparison as a mechanism of change in PA apps (eg, randomized controlled trials, meta-analyses, and dismantling studies) to answer the question of whether, for whom, or under what circumstances social comparison features of apps produce positive changes in PA. Such research is critical to advance our basic understanding of comparison processes and their utility as BCTs, as is further information about within-person variability in comparison preferences and responses. This information would inform the necessary or sufficient social comparison features of PA apps needed for a successful intervention. To what extent our findings and conclusions apply beyond PA promotion (alone or in the context of weight control) to such health behaviors as smoking cessation or skin cancer prevention [74,75] remains to be addressed in future research.

### Conclusions

This review documents that social comparison is frequently identified as a potential mechanism of behavior change in smartphone apps designed to promote PA, on par with mechanisms such as social networking (broadly defined). Behavioral modeling, which is considered in some reviews as a means of inducing social comparison, was the only comparison-related mechanism to appear in more reviews of PA apps than social comparison (as explicitly differentiated from other processes). Our findings highlight the need for careful consideration of social processes as behavior change mechanisms in app design and evaluation. Considerable gaps currently exist between theory and evidence relevant to social comparison and its implementation in PA apps. Greater attention to individual differences, dynamic responses, relevant PA dimensions, and comparison preferences and the potential to tailor apps on the basis of these characteristics may meaningfully improve the effectiveness of existing PA promotion apps.

## Abbreviations

BCT: behavior change technique
mHealth: mobile health
PA: physical activity
PRISMA: Preferred Reporting Items for Systematic Reviews and Meta-Analyses
SC: social comparison
